# Into the darkness of the microbial dark matter *in situ* activities through expression profiles of *Patescibacteria* populations

**DOI:** 10.3389/fmicb.2022.1073483

**Published:** 2023-01-09

**Authors:** Adrien Vigneron, Perrine Cruaud, Rémy Guyoneaud, Marisol Goñi-Urriza

**Affiliations:** ^1^IBEAS, Université de Pau et des Pays de l'Adour, E2S UPPA, CNRS, IPREM, Pau, France; ^2^Independent Researcher, Lourenties, France

**Keywords:** CPR bacteria, Parcubacteria, MAGs, metatranscriptomic, microbial mats, genome-centric metatranscriptomic, ecophysiology

## Abstract

*Patescibacteria* form a highly diverse and widespread superphylum of uncultured microorganisms representing a third of the global microbial diversity. Most of our knowledge on *Patescibacteria* putative physiology relies on metagenomic mining and metagenome-assembled genomes, but the *in situ* activities and the ecophysiology of these microorganisms have been rarely explored, leaving the role of *Patescibacteria* in ecosystems elusive. Using a genome-centric metatranscriptomic approach, we analyzed the diel and seasonal gene transcription profiles of 18 *Patescibacteria* populations in brackish microbial mats to test whether our understanding of *Patescibacteria* metabolism allows the extrapolation of their *in situ* activities. Although our results revealed a circadian cycle in *Patescibacteria* activities, a strong streamlined genetic expression characterized the *Patescibacteria* populations. This result has a major consequence for the extrapolation of their physiology and environmental function since most transcribed genes were uncharacterized, indicating that the ecophysiology of *Patescibacteria* cannot be yet reliably predicted from genomic data.

## Introduction

Metagenomic sequencing has significantly expanded our knowledge of microbial diversity in various environments (Parks et al., 2017). This approach has notably revealed the “microbial dark matter,” gathering a large fraction of the global microbial diversity that escaped culture and PCR-based surveys (Rinke et al., 2013; Nobu et al., 2015). The candidate phyla radiation (CPR), also known as the *Patescibacteria* superphylum, has emerged from this microbial dark matter. Members of this superphylum have now been detected almost ubiquitously in natural environments, including in human-related samples (Castelle et al., 2018), representing a significant proportion (7.5%) of the communities in most ecosystems (Beam et al., 2020). However, when the filtration strategy of aquatic samples included a size fraction lower than 0.22 μm, the relative proportion of *Patescibacteria* lineages frequently exceeded those of other bacteria in diversity surveys, suggesting that the abundance of ultra-small *Patescibacteria* is frequently underestimated (Luef et al., 2015; Anantharaman et al., 2016; Danczak et al., 2017; Vigneron et al., 2020; Chaudhari et al., 2021). Most of our knowledge about these microorganisms derives from population genomes recovered from metagenomic datasets (Brown et al., 2015; Hug et al., 2016; Castelle and Banfield, 2018; Tian et al., 2020). With more than 5000 draft genomes of *Patescibacteria* in public databases, the metabolic repertoire of the *Patescibacteria* members is rapidly expanding. Shared features of *Patescibacteria* include a small genome size with limited known biosynthetic and metabolic pathways (Castelle and Banfield, 2018; Tian et al., 2020). Therefore, a potential episymbiotic and fermentative lifestyle is frequently assumed for the *Patescibacteria* (Anantharaman et al., 2016; Castelle and Banfield, 2018; Castelle et al., 2018). While the majority of this superphylum remains uncultivated, some representatives of the *Saccharimonadia* group (previously known as TM7) were found to be episymbiotic of actinobacterial cells (He et al., 2015; Bor et al., 2020; Murugkar et al., 2020). Few members of the *Gracilibacteria* were also characterized as predatory bacteria of phototrophic *Gammaproteobacteria* (Moreira et al., 2021; Yakimov et al., 2021), and members of the *Yanofskybacteria* have been observed in the vicinity of methanogenic archaea (Kuroda et al., 2022), supporting the general assumption of a host-associated lifestyle for *Patescibacteria*. However, evidence for a host-dependent lifestyle is currently limited to the few characterized lineages, and the putative metabolism of most of the *Patescibacteria* lineages remains misunderstood. Free-living *Patescibacteria* were also observed, suggesting a broad range of potential life strategies in this phylum (Chiriac et al., 2022). However, these results might have pictured a “free-floating” stage in the life cycle of episymbiotic/parasitic *Patescibacteria*, as observed with *Saccharibacteria* (Bor et al., 2018). Since essential functions, such as genetic information processing, including nucleotide metabolic processes, were conserved, the reduced genome size observed in *Patescibacteria* could also be associated with an evolutionary streamlining to reduce functional redundancy and energy waste for the free-living bacteria (Tian et al., 2020). Consistently, a limited catabolic potential has been detected for different Patescibacteria lineages, suggesting a potential niche partitioning based on different carbohydrate utilization (Danczak et al., 2017; Vigneron et al., 2020). However, due to the lack of cultured representatives and ecophysiological studies, these metabolic hypotheses predicted from genomic data have not been tested, leaving the activity and ecological roles of these microorganisms elusive.

Taking benefit of an extensive metagenomic (370 Gb of reads) and metatranscriptomic (300 Gb of rRNA-depleted reads) dataset (SRP063590) covering sample heterogeneity as well as seasonal and diel variability of two microbial mats located in brackish lagoons (Salins du Lion and Etang de Berre, France) (Vigneron et al., 2021), we use a genome-centric metatranscriptomic approach to investigate *in situ* activities of *Patescibacteria* lineages. More specifically, we tested if our current understanding of the *Patescibacteria* genomic repertoire allowed the extrapolation of their putative activities.

## Materials and methods

### Metagenomic and metatranscriptomic samples

In this study, we analyzed metagenomic and metatranscriptomic sequences recovered from samples collected in two coastal microbial mats developing in neighboring brackish lagoons located at l'Etang de Berre (EDB) and Salins du Lion (SL). These microbial mats were strongly stratified with steep gradients of oxygen, nitrogen, and sulfur compounds throughout the thickness of the mats, leading to both aerobic and anoxic niches (Aubé et al., 2020; Vigneron et al., 2021). As previously described (Aubé et al., 2020), the sequenced DNA and RNA originating from 30 microbial mat samples were collected in triplicate in September 2011, April 2012, and September 2012 during both daytime (4 PM) and nighttime (4 AM). Nucleic acid extraction and library preparation, including ribosomal RNA depletion of the metatranscriptomes, were previously described (Aubé et al., 2020). Illumina HiSeq sequencing of the libraries led to a total of 670 Gb of sequencing reads (370 GB of metagenomes and 300 GB of rRNA-depleted metatranscriptomes). Datasets were quality filtered using the Trimmomatic v.0.39 tool keeping both R1 and R2 reads when reads overlapped (Bolger et al., 2014). The 16S rRNA reads were isolated from the metagenomic reads using Infernal v.1.1.4 (Nawrocki and Eddy, 2013), and their taxonomic assignments were performed with Mothur (Schloss et al., 2009) using BLAST against Silva database release 138 as reference (Pruesse et al., 2007). Since metagenomic 16S rRNA reads were only 100-bp long and spanned various regions of the 16S rRNA gene, taxonomic assignments were limited to the genus level and above.

### Binning and functional characterization

For metagenome-assembled genome (MAG) reconstruction, all quality-filtered sequences were pooled and co-assembled using MEGAHIT v.1.2.9 (Li et al., 2015), as previously described (Vigneron et al., 2021). Read coverage of the contigs against all metagenomic datasets was carried out using bwa-mem v.0.7.17 (http://bio-bwa.sourceforge.net), and the binning of the contigs longer than 2000 bp was carried out by MetaBAT-2 (Kang et al., 2015). Coding density, completeness, and contamination levels of the MAGs were then evaluated using CheckM v.1.1.2 (Parks et al., 2015) with cpr_43_markers.hmm files that provide a better estimation of CPR bacteria completeness (Parks et al., 2015). Taxonomic affiliation of the MAGs was performed using the GTDB_Tk classify workflow (GTDB-tk release 207) with genomes containing a minimum of 40% of the bacterial marker genes (Chaumeil et al., 2020). In addition, a phylogenomic tree was constructed based on the concatenated alignment of all ribosomal protein sequences detected in the MAGs (rpL2, 3, 4, 5, 6, 14, 15, 16, 18, 22, 24, rpS3, 6, 8, 10, 17, 19) using metabolisHMM (McDaniel et al., 2020). Ribosomal proteins were aligned using mafft v.7.471 (Katoh et al., 2002), and a phylogenetic tree of the concatenated alignment (62 sequences with 3729 amino acid positions) was produced using FastTree2 with 1000 bootstraps (Price et al., 2010). Open reading frames (ORFs) were identified using Prodigal (Hyatt et al., 2010) with coding table 11, except for *Gracilibacteria* MAGs that required coding table 23 (Campbell et al., 2013). ORFs were then compared against COG (Galperin et al., 2021), TIGRfam (Haft et al., 2003), and KEGG (Kanehisa et al., 2016) databases on IMG/MER platform using IMG annotation pipeline v.5.0.19 (Markowitz et al., 2009), leading to 5.7 x10^6^ genes coding for proteins with the product name (58.96% of the genes). ORFs were also compared against the CAZY database using dbcan2, and glycoside hydrolase genes were analyzed to infer the catabolic potential (Zhang et al., 2018). The type of ribulose-1,5-bisphosphate carboxylase (Rubisco) protein was determined using publicly available HMM profiles of the different Rubisco categories (Anantharaman et al., 2016). Metatranscriptomic reads passing quality filtration (4.12 ± 0.7 x 10^7^ reads passing quality filtration per sample for a total of 1.24 x 10^9^ metatranscriptomic reads) were normalized and then mapped against all ORF identified in the MAGs using bwa-mem with a default value to determine the expression level of each gene under all tested conditions. To limit computing time, the mapping of the metatranscriptomic reads was performed only against samples where MAGs were recovered. For the comparison with other phyla, only MAGs with a coverage similar to *Patescibacteria* MAGs were analyzed, and only phylum with at least eight representative MAGs were included.

## Results and discussion

**Figure 1:**
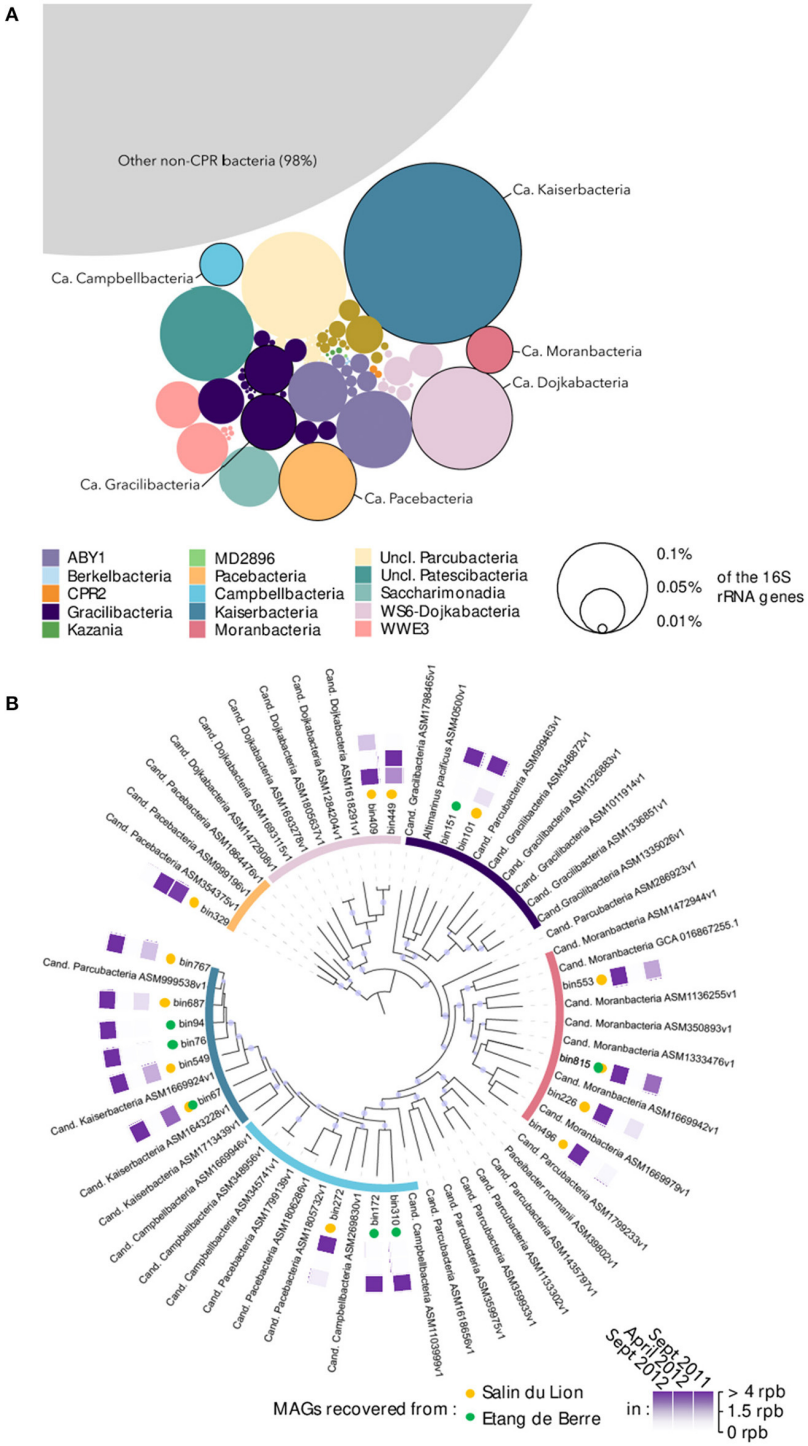
**(A)** Overall *Patescibacteria* community composition in the microbial mats based on 16S rRNA genes identified in the metagenomic dataset. The size of the bubbles represents the average relative proportion of each lineage across all samples. Circled bubbles indicate lineages with representative MAGs. **(B)** Phylogenomic tree of the *Patescibacteria* MAGs recovered in the microbial mats. The tree was constructed based on concatenated ribosomal protein genes alignment with the closest representative genomes available on NCBI. The purple points in the branches of the tree represent bootstrap values >0.8. The inner circle is color coded as in **(A)**. Yellow/green dots indicate the origin of the MAG, respectively. Purple squares are heat maps indicating the season in which MAGs were detected based on the coverage of the MAGs (rpb, reads per bases). For example, bin767 was detected in September 2011, not detected in April 2012, and strongly detected again in September 2012.

The microbial community composition of the two microbial mats was characterized using 16S rRNA genes recovered from the 30 metagenomes. Overall, *Patescibacteria* constituted a minor but diverse fraction of the microbial mats community, representing in total 2% of the 16S rRNA reads from the metagenomic dataset (Figure 1A). Nonetheless, spatial and seasonal variations in the *Patescibacteria* community composition were detected (Supplementary Figure 1), suggesting rapid responses to environmental conditions. After co-assembly of the metagenomic datasets and binning of the contigs longer than 2000 bp, 18 MAGs (average completeness: 90 ± 10%) over the 407 MAGs recovered from the mats were affiliated to *Patescibacteria*, including representatives of the Candidatus *Pacebacteria, Dojkabacteria, Gracilibacteria, Moranbacteria, Campbellbacteria*, and *Kaiserbacteria* phyla (Figure 1B), that were identified as dominant *Patescibacteria* by 16S rRNA gene analysis. The coverage of the *Patescibacteria* MAGs averaged at 2.75 reads per base in contigs (max: 12.68, min: 0.85), which is below the average coverage of the 407 recovered MAGs (3.5) but higher than the median value (1.62). This result suggests that although *Patescibacteria* represented a small fraction of the community, each individual population has a similar relative abundance to most of the other populations found in the microbial mats and could, therefore, play an important role in these ecosystems.

### Genomic composition of Patescibacteria

**Figure 2:**
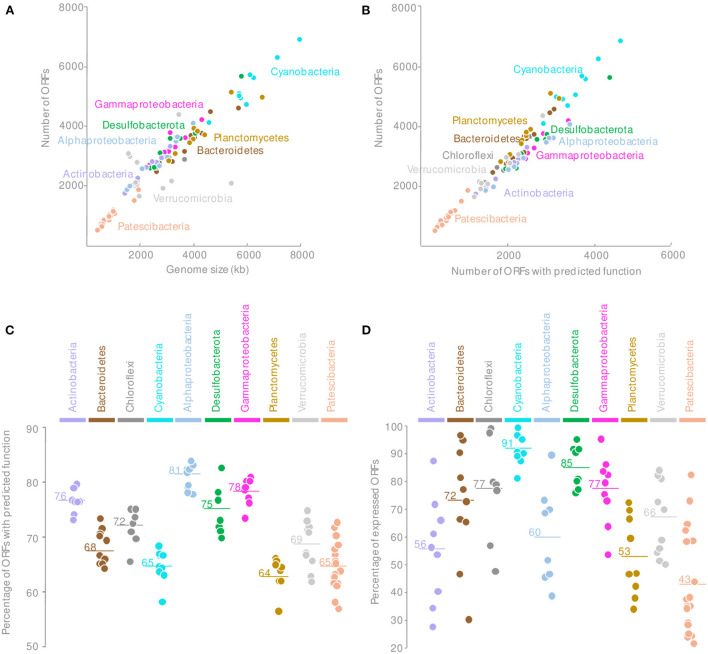
Comparison of the MAGs genomic content of the *Patescibacteria* with other microbial phyla recovered from the microbial mats with **(A)** the number of ORFs by the estimated genome size, **(B)** the relationship between the total number of ORFs and the number of ORFs with predicted function; **(C)** the percentage of ORFs with predicted functions, and **(D)** the percentage of expressed ORFs for each major phyla of the microbial mats. Horizontal lines indicate the mean value.

Consistently with publicly available genomes, the *Patescibacteria* MAGs were reduced in size, and an average of 940 ORFs per MAG were identified, indicating a lower genomic content for a similar completeness level compared with other phyla found in the microbial mats (Figure 2A and Supplementary Figure 2). Comparison against Kegg (Kanehisa et al., 2016), COG (Galperin et al., 2021), TIGRFAM (Haft et al., 2003), and CAZy (Cantarel et al., 2009) databases assigned together a predicted function for 65 ± 4% of the proteins encoded in ORFs (Supplementary Figure 3), which are lower than the average number of proteins with a predicted function for the entire microbial mat community (70%) but comparable to *Planctomycetes* or *Cyanobacteria* (Figures 2B, C). Although some key functional genes might not have been detected due to the incompleteness of some MAGs and sequence divergence, the overall *Patescibacteria* metabolic potential, inferred from the identified proteins, was consistent with previously published genomes (Castelle and Banfield, 2018).

**Figure 3:**
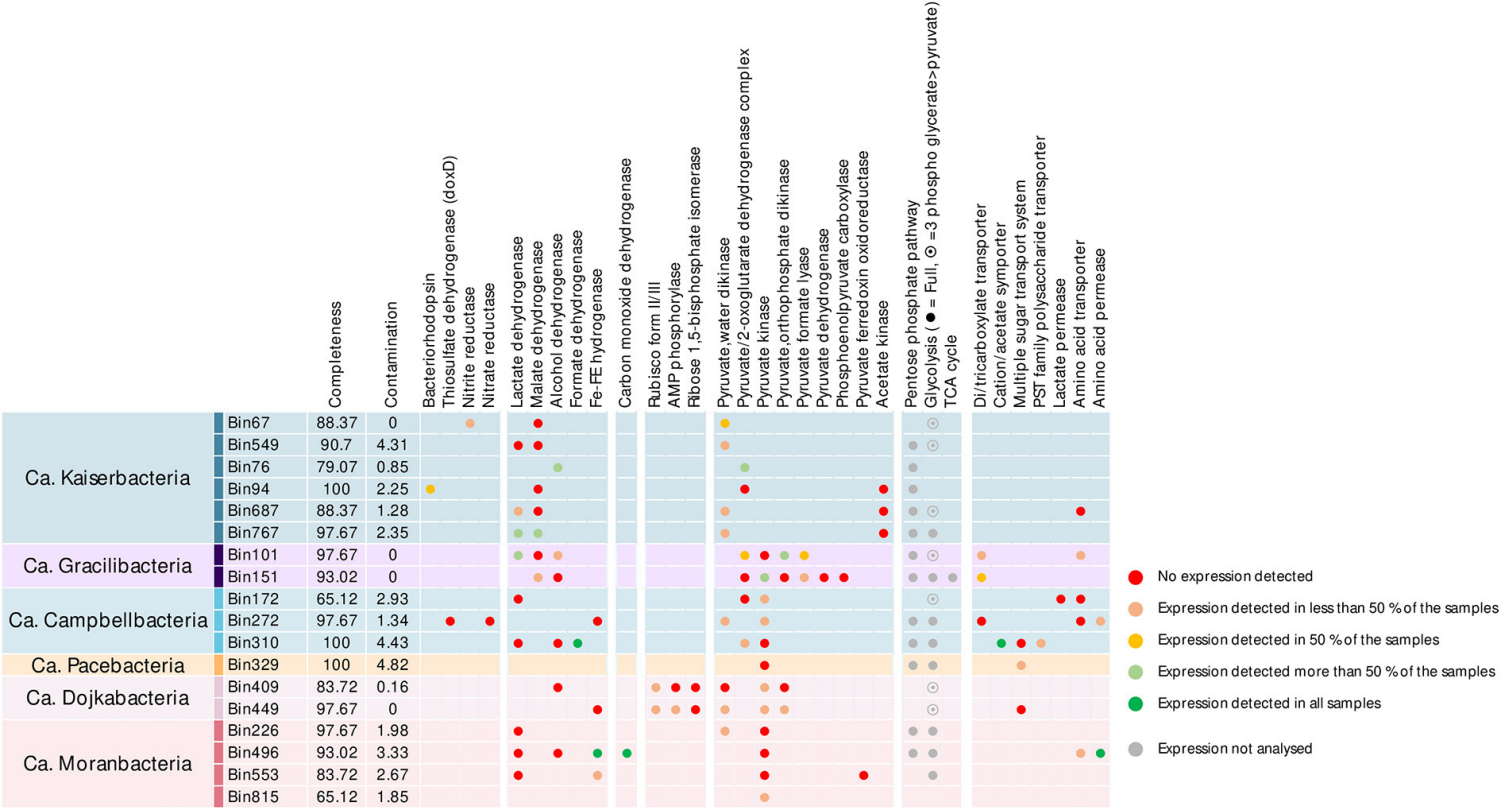
Metabolic potential and activities identified in the Patescibacteria MAGs of the microbial mats. Completeness and contamination percentages were estimated using CheckM with cpr_43_markers hmm profiles. Dots indicate the detection of the gene in the MAGs and are color-coded based on the identification of corresponding transcripts in the metatranscriptomic dataset. Due to the high number of genes involved, the expression level of complex metabolic pathways (pentose phosphate pathway, glycolysis/neoglucogenesis, and tricarboxylic acid cycle) was not estimated (gray dots).

Most of the MAGs included the pentose phosphate pathway and the full or terminal part of glycolysis/gluconeogenesis (Figure 3). Genes involved in the AMP salvage pathway (Wrighton et al., 2016), including gene coding for the type II/III ribulose-1,5-bisphosphate carboxylase (Rubisco), the AMP phosphorylase, and the ribose-1,5-bisphosphate isomerase were identified in *Ca. Dojkabacteria* MAGs, supporting the potential for ribose degradation in some *Patescibacteria* phyla (Wrighton et al., 2016). While forms I and II of the Rubisco can fix atmospheric carbon dioxide during primary production *via* the Calvin–Benson–Bassham cycle, forms II/III and III, found primarily in archaea, enable light-independent CO_2_ incorporation into sugars derived from nucleosides (Sato et al., 2007). Previously detected in various Patecibacteria lineages, including other *Dojkabacteria* (Castelle et al., 2018) and DPANN *Archaea* (Vigneron et al., 2022), enzymes coded by these genes catalyze the conversion of nucleoside to glycerate-3P, which is then incorporated through the terminal part of glycolysis. Genes of proteins converting phosphoenolpyruvate generated by the glycolysis or the AMP salvage pathway into pyruvate were detected in most of the MAGs. Genes coding for Fe–Fe hydrogenase and lactate, malate, formate, and alcohol dehydrogenases were detected in the MAGs, providing various pathways for pyruvate fermentation (Figure 3) and supporting a basic metabolism centered on simple metabolites (Tian et al., 2020). A limited number of genes coding for transporters were detected in the MAGs, as previously reported (Tian et al., 2020; Vigneron et al., 2020). Transport systems for simple sugars, polysaccharides, amino acids, and volatile fatty acids were identified, notably in *Campbellbacteria*, suggesting a potential uptake of these compounds from the environment or a potential host. Genes encoding di-/tri-carboxylate transporters, allowing the uptake of intermediates of the tricarboxylic acid and Krebs cycles (e.g., citrate, fumarate, succinate, and malate), were also identified in *Gracilibacteria* MAGs (Figure 3). These MAGs also include oxaloacetate-decarboxylating malate dehydrogenase genes to convert these substrates into pyruvate, and even the TCA cycle for bin151, supporting the assimilation of di-/tricarboxylates produced by a potential host or excreted in the microbial mat matrix by other microorganisms (Krom et al., 2003).

Limited catabolic capabilities were identified among the different *Patescibacteria* lineages (max: nine glycoside hydrolase genes per MAG, Supplementary Figure 4). Consistently with previous investigations, potential substrates included cellulose and its components (GH3, GH5, GH39, and GH78), amylose (GH13, GH15, and GH57), chitin (GH18), polysaccharides (GH28, GH65, and GH100), and peptidoglycan (GH23 and GH108) (Danczak et al., 2017; Vigneron et al., 2019). Laccases and peroxidases genes were also identified in few MAGs, suggesting that some *Patescibacteria* lineages could also degrade lignin.

Alternative energy-conservating pathways were scarce in *Patescibacteria* MAGs but included genes coding for bacteriorhodopsin, thiosulfate dehydrogenase, and nitrate and nitrite reductases, supporting previous results (León-Zayas et al., 2017; Jaffe et al., 2021). The microbial mats are characterized by steep gradients of light, oxygen, nitrogen, and sulfur compounds (Aubé et al., 2016), and sulfur and nitrogen cycles have been previously identified as major processes in these mats (Aubé et al., 2020), supporting such metabolism across the thickness of the mats.

In addition, the large subunit of the aerobic carbon monoxide dehydrogenase form II (CoxL) was identified in one *Moranbacteria* MAG (Figure 3), suggesting the potential for CO oxidation. Aerobic and anaerobic carbon monoxide dehydrogenase genes have not been reported in *Patescibacteria* so far (Adam et al., 2018); however, the catalytic site motif AYRGAGR, and HGRDH, HSHGQ motifs that occur consistently in characterized form II CoxL were detected in the sequence (King and Weber, 2007), supporting the identification of the gene. A comparison of the sequence against the NCBI database indicated that the sequence shared 74% similarity with the carbon monoxide dehydrogenase gene from an uncultured marine organism and 67% similarity with the carbon monoxide dehydrogenase gene of *Rokubacteria* MAGs. Nonetheless, other subunits of the complex were not identified based on sequence similarity. However, it has been suggested that the lack of other subunits may indicate that this enzyme could act on substrates other than CO (King and Weber, 2007). Therefore, further work is needed to characterize the activity and distribution of putative aerobic carbon monoxide dehydrogenase in *Patescibacteria*.

### *In situ* activities of Patescibacteria

**Figure 4:**
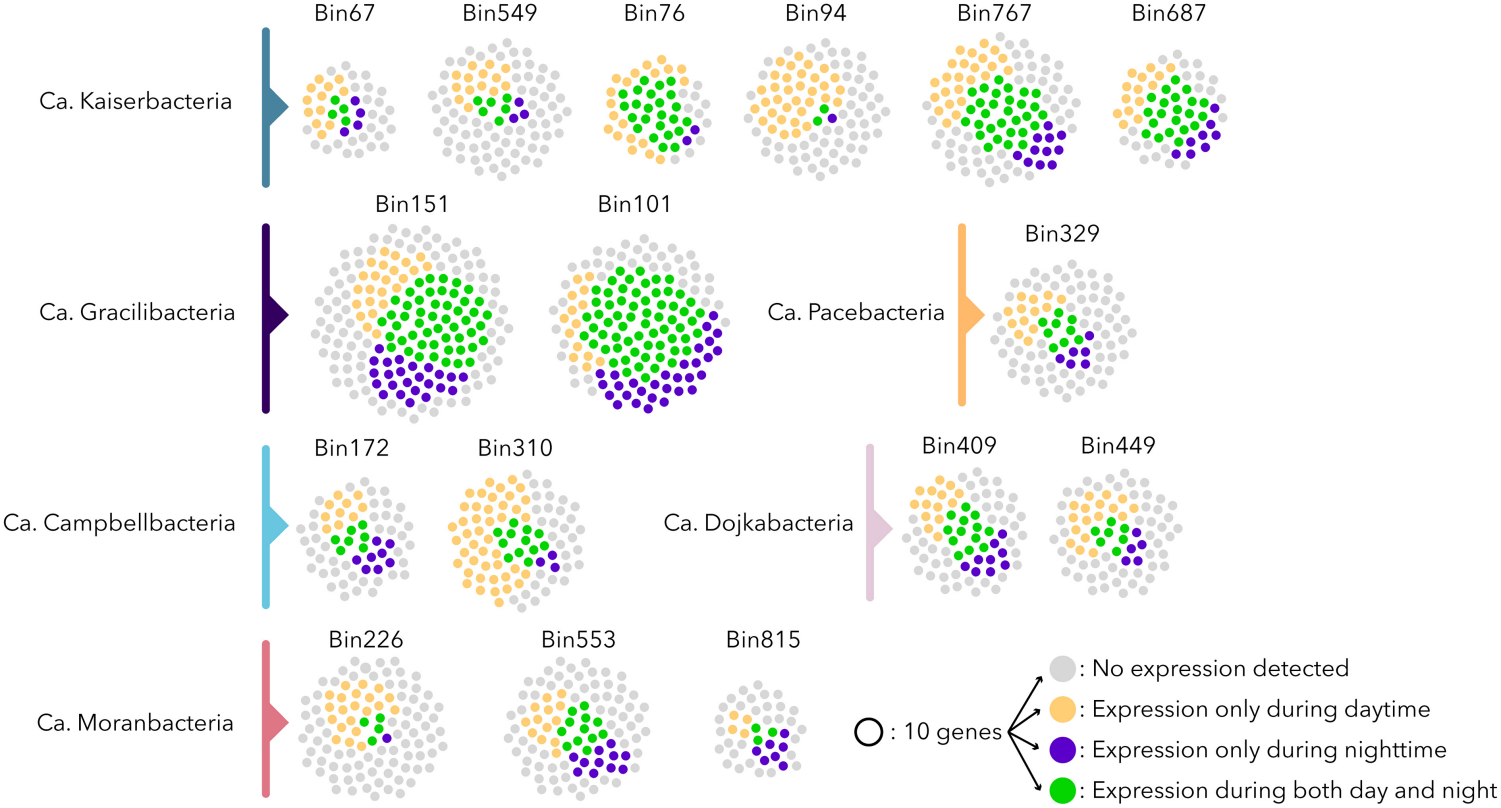
Expression profile of the *Patescibacteria* populations. Each point represents 10 ORFs; gray points indicate no expression detected, and yellow and blue points represent genes expressed only during daytime and nighttime, respectively. Green points represent genes expressed during both daytime and nighttime. Bin272 and bin496 were omitted since they were recovered from samples collected in September 2011 without day/night samples.

The extended metatranscriptomic dataset (4.12 ± 0.7 x 10^7^ reads passing quality filtration per sample for a total of 1.24 x 10^9^ metatranscriptomic reads) that covered the sample heterogeneity, as well as the seasonal (April and September) and diel variations of the microbial mats, was mapped against *Patescibacteria* ORFs (Supplementary Table 2). An analysis of the expression level of ribosomal protein genes, DNA polymerases, and the number of expressed genes indicated differential activity levels for the *Patescibacteria* populations of the microbial mats (Figure 4). Only 32% of the expressed genes were transcribed under both day and night conditions, suggesting contrasted diel activities in *Patescibacteria* (Figure 4 and Supplementary Table 2). Apart from Ca. *Gracilibacteria* that showed higher nighttime activity, all other *Patescibacteria* populations expressed on average three times more genes during daytime than nighttime, with a maximum of 19 times for a Ca. *Kaiserbacteria* (bin94) population with a bacteriorhodopsin-like gene expressed only during daytime. Bacteriorhodopsin genes have been previously identified in freshwater *Patescibacteria* (Chiriac et al., 2022; Jaffe et al., 2022), and proton transport assays confirmed the light-inducted proton translocation role of this rhodopsin (Jaffe et al., 2022), supporting the ability to produce energy from light for some *Patescibacteria* lineages. This result suggests that some *Patescibacteria* activity might be influenced directly by light. Water temperature, that fluctuated from 21.5°C during the day to 13°C at night, as well as dissolved oxygen (day: 10.9 mg/L, night: 4.6 mg/L) and redox potential (day: −200 Mv, night: −295 Mv) that are both linked to the activity of light harvesting microorganisms, also varied between daytime and nighttime and could also potentially shape *Patescibacteria* activities. In addition, activities of other diel microorganisms, such as photosynthetic *Cyanobacteria* or anoxygenic phototrophs that represent a large fraction of the microbial mat community (Vigneron et al., 2021), might also influence the gene expression pattern of *Patescibacteria* that rely on these lineages to fulfill their metabolic dependences.

Surprisingly, only 43.4 ± 18% of the *Patescibacteria* ORFs were expressed in at least one sample regardless of the estimated level of activity (Figure 2D). This proportion of transcribed ORFs is lower than for any other MAGs from other phyla recovered from the same samples and with similar coverage (Figure 2D). These results indicated that although *Patescibacteria* lineages harbored a reduced genomic content, a large fraction of their genomes were not expressed under the contrasting environmental conditions of our sampling campaigns. These results might indicate a rapid transcriptomic control, as illustrated by the dial shift, for a fine-tuning of their activities. This streamlined activity could represent an ecological benefit in the context of the Black Queen Hypothesis that characterizes the *Patescibacteria* genome evolution (Tian et al., 2020). In a complex and dense microbial ecosystem where by- and end-products of catabolic reactions are probably abundant, *Patescibacteria* populations could save their energy by streamlining their genetic expression and benefit from catabolic products available in the microbial mat matrix. This could also illustrate a rapid synchronization of the *Patescibacteria* to the activity of their potential host. Alternatively, this result might indicate that the genome reduction process is still ongoing. However, the coding density of the *Patescibacteria* MAGs was similar to the rest of the microbial community (89 ± 3%), providing little support for gene inactivation and erosion (Liu et al., 2004).

Among the expressed genes, an average of 66.7% (206 ± 132 ORFs per MAG) encoded proteins with a predicted function (Supplementary Figure 3 and Supplementary dataset). However, only 13% of the genes used for the metabolic characterization of the MAGs were expressed in more than 50% of the samples, and 52% of the samples were not expressed in any of the samples (Figure 3 and Supplementary Figure 4). Furthermore, 45 ± 19% of the most expressed genes in MAGs were not associated with a known function (Supplementary Figure 2), confirming that substantial activities remain uncharacterized. These results have major consequences for the extrapolation of the *Patescibacteria* metabolism and environmental function. For instance, transcripts of alcohol, lactate, or malate dehydrogenases were rarely detected, challenging the fermentative metabolism assumed from the detection of these genes in the genomic data. Although we cannot exclude that some transcripts were not sequenced, these results suggest that essential genes and pathways remain uncharacterized in the microbial dark matter or unidentified due to overly divergent sequences compared with existing databases. This indicates that *in situ* activities of *Patescibacteria* could hardly be inferred from metagenomic data alone and that activity-based and cultivation data are mandatory.

## Data availability statement

The datasets presented in this study can be found in NCBI Genbank under the Bioproject accession number SRP063590.

## Author contributions

AV and PC analyzed the data, designed the figures, and wrote the manuscript with contributions and corrections from RG and MG-U. AV, RG, and MG-U designed the study. All authors contributed to the article and approved the submitted version.
