# Supplementary material for: Into the darkness of the microbial dark matter in situ activities through expression profiles of Patescibacteria populations
Source: Front Microbiol. 2023 Jan 9;13:1073483. doi: 10.3389/fmicb.2022.1073483 (PMC9868632; doi:10.3389/fmicb.2022.1073483)
Supplement: Supplementary file 3 [file Data_Sheet_1.pdf]

Supplementary Material

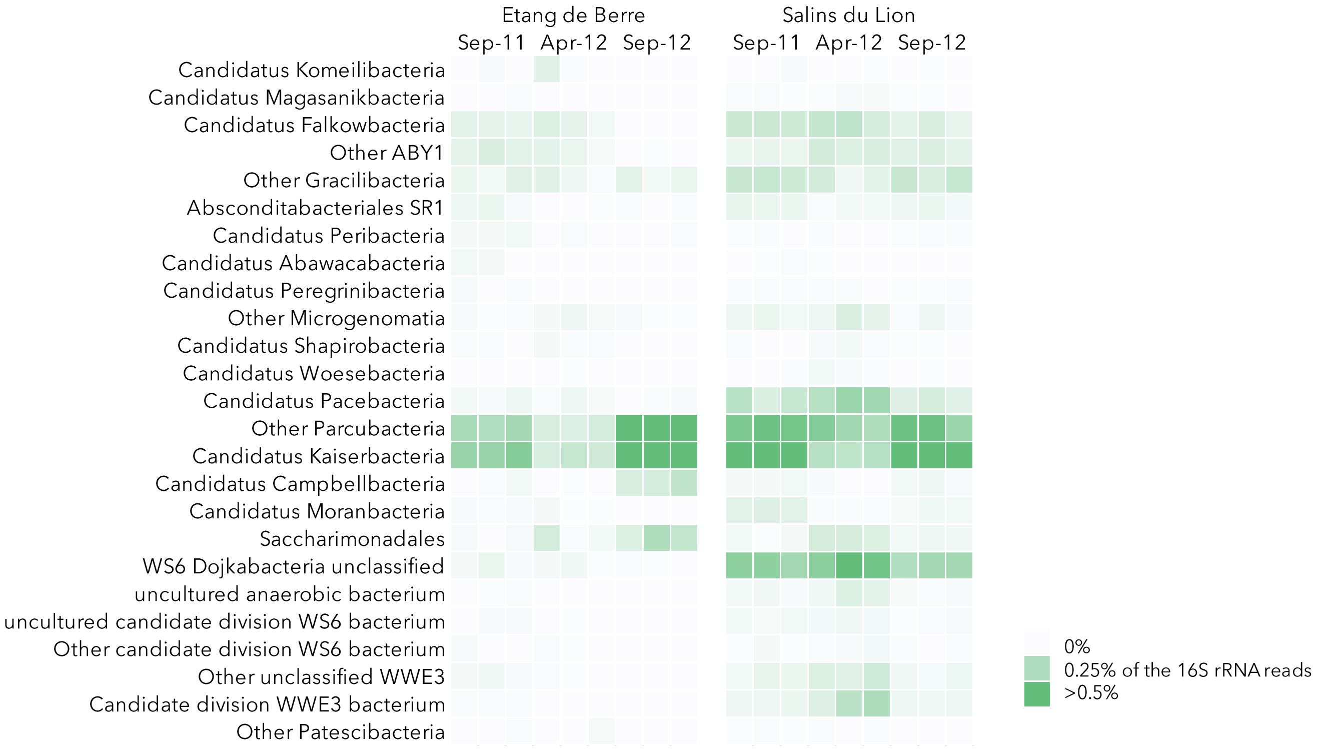

Supplementary Figure 1 Patescibacteria community composition in all microbial mat samples, based on 16S rRNA genes identified in the metagenomic dataset

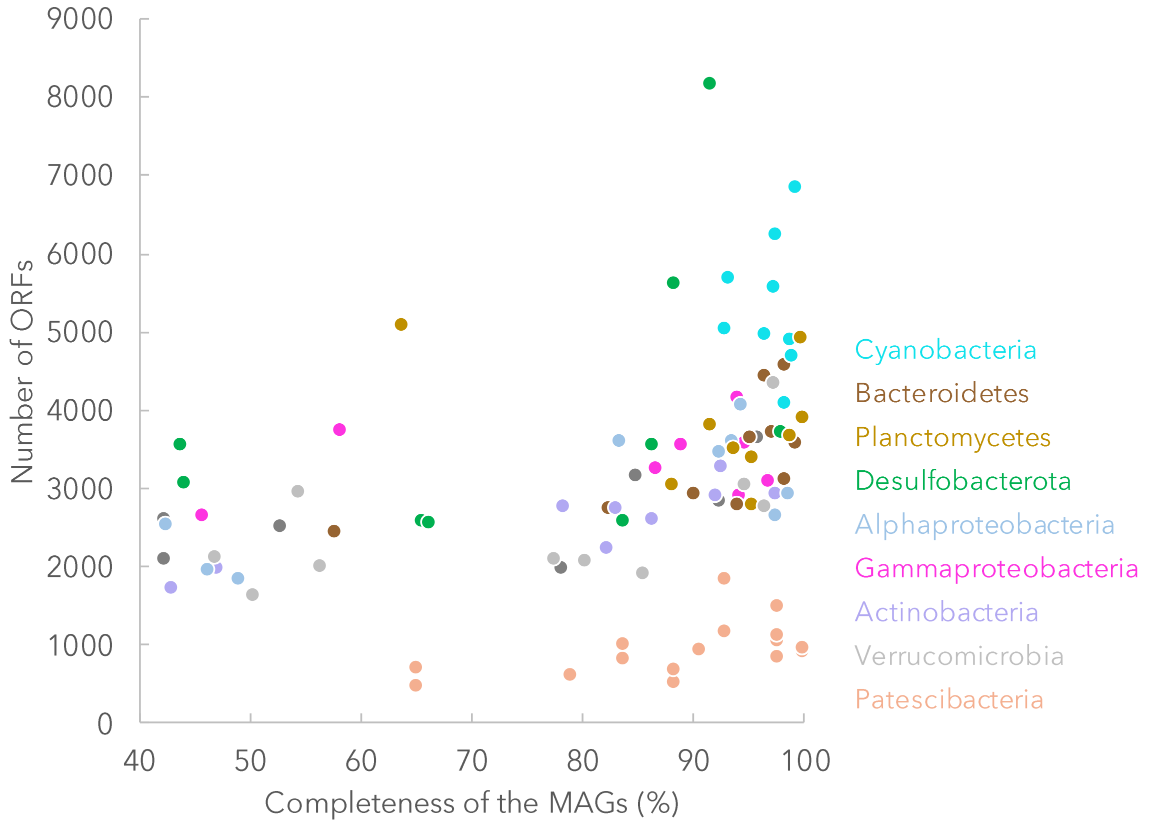

Supplementary Figure 2 Comparison of the MAGs genomic content of the *Patescibacteria* with other microbial phyla recovered from the microbial mats with the number of ORFs detected in the MAGs by the estimated completeness of each MAGs.

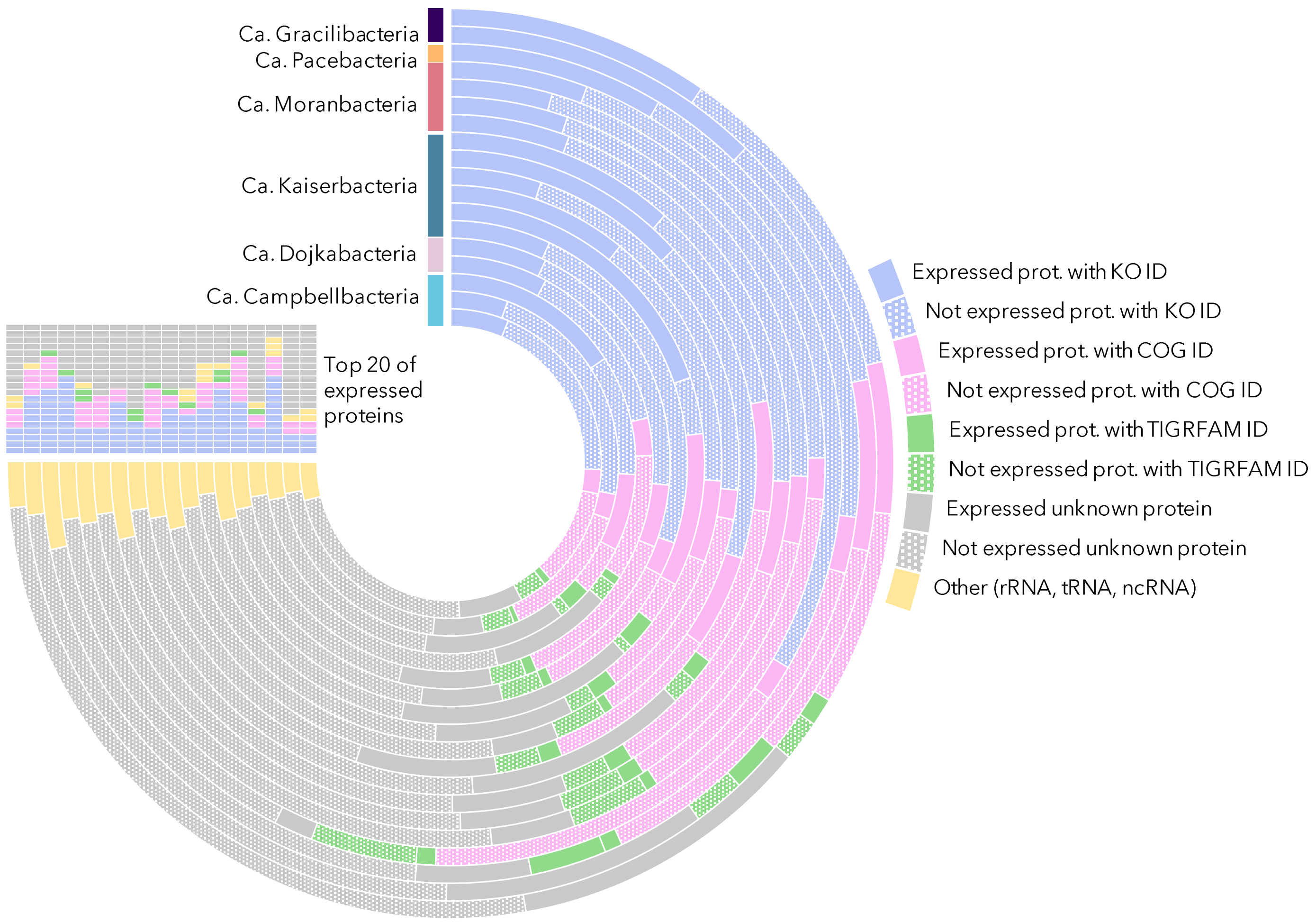

Supplementary Figure 3. Cumulated analysis of the genomic content and expression profile of the *Patescibacteria* MAGs. Blue shades indicate the proportion of genes with product name assigned with Kegg database, pink shades indicate additional ORFs identified with COG database and green shades additional ORFS identified with TIGRFAM database for all ORFs and the most expressed ORFs (top 20) of the MAGs. Grey shades represent the proportion of genes with no identified function and yellow represent tRNA, siRNA and miRNA. Dotted fractions indicate the proportion of ORFs without corresponding transcript in the whole metatranscriptomic dataset.

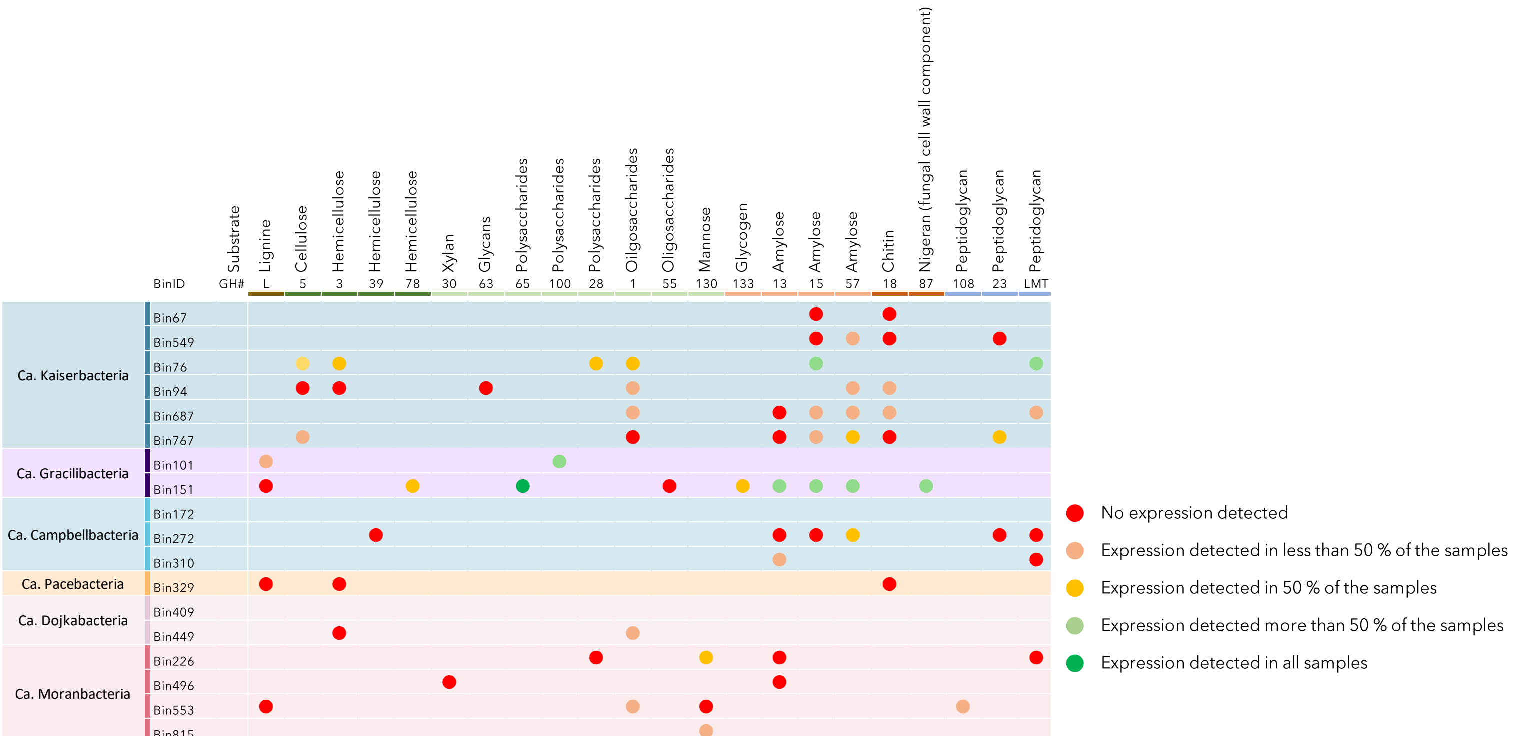

Supplementary Figure 4. Catabolic potential and activities identified in the *Patescibacteria* MAGs based on the detection of genes and transcripts of carbohydrate activating enzymes. Dots indicate the detection of the gene in the MAGs and are color coded based on the identification of corresponding transcripts in the metatranscriptomic dataset.

Supplementary Dataset. Detailed list of genes identified in the *Patescibacteria* MAGs
